# LncRNA NRON down-regulates lncRNA snaR and inhibits cancer cell proliferation in TNBC

**DOI:** 10.1042/BSR20190468

**Published:** 2019-05-14

**Authors:** Limin Niu, Qingxia Fan, Min Yan, Liuxing Wang

**Affiliations:** 1 The First Affiliated Hospital of Zhengzhou University, Zhengzhou City, Henan Province 450052, P.R. China; 2 the Affiliated Cancer Hospital of Zhengzhou University, Zhengzhou City, Henan Province 450008, P.R. China; 3 Department of Breast Surgery, Henan Cancer Hospital, Zhengzhou City, Henan Province 450008, P.R. China

**Keywords:** triple negative breast cancer, lncRNA NRON, lncRNA snaR

## Abstract

NRON mediates the degradation of tat protein to participate in HIV-1 infection. Interestingly, our study observed the down-regulation of NRON in triple-negative breast cancer (TNBC) tissues compared with paired adjacent healthy tissues. In contrast, lncRNA snaR was up-regulated in TNBC tissues and was inversely correlated with NRON. Expression levels of snaR increased, while expression levels of NRON decreased along with the increase of clinical stages. The snaR overexpression resulted in promoted cancer cell proliferation but did not significantly affect NRON expression. NRON overexpression inhibited cancer cell proliferation and down-regulated snaR. The snaR overexpression reduced the effects of NRON overexpression. We therefore conclude that NRON may down-regulate lncRNA snaR to inhibit cancer cell proliferation in TNBC.

## Introduction

Triple-negative breast cancer (TNBC) is major subtype of breast cancer than is characterized by its aggressive nature [1]. Triple-negative refers to the absence of expression of human epidermal growth factor receptor 2 (HER2), progesterone receptor (PR), and estrogen receptor (ER) [2]. TNBC patients are prone to develop distant metastases, thus leading to unacceptably high mortality rate [3]. In addition, TNBC has a tendency to affect young females and responds poorly to almost all available targeted therapy [4]. Therefore, prevention and treatment of TNBC are of great clinical significance. However, the molecular mechanism of the aggressive nature of TNBC is still hardly known, leading to failures of clinical treatment and high recurrence rate [5].

Oncology studies used to focus on oncogenes and tumor suppressors [6], and the complicated pathogenesis of cancer may require the participants of other internal factors, such as lncRNAs (>200 nt), which are RNA transcripts lacking protein-coding capacity but participate in diverse physiological and pathological processes, such as cancer development by regulating gene expression [7,8]. To date, the function of most lncRNAs is still unknown, which hinders the application of lncRNAs in cancer prediction and treatment. A recent study reported a novel gene, named NRON, which can regulate HIV-1 infection by inducing the degradation of tat protein [9]. Interestingly, our preliminary deep sequencing data revealed that NRON was down-regulated in TNBC, but not in other types of breast cancer, and was inversely correlated with snaR, which plays oncogenic roles in TNBC [10]. We therefore investigated the potential involvement of NRON in TNBC and analyzed its relationship with snaR.

## Materials and methods

### Patients’ specimens

All TNBC and adjacent non-cancer tissue specimens were from 70 patients with TNBC before any therapies. All specimens were confirmed by at least three experienced pathologists. All the patients were diagnosed through histological biopsy in the First Affiliated Hospital of Zhengzhou University Hospital and Henan Cancer Hospital between June 2016 and June 2018. Non-cancer tissues were obtained during biopsy. Inclusion criteria: (1) TNBC patients who were diagnosed for the first time; (2) no previous history of maligancy was observed; (3) no therapies received before the present study. Exclusion criteira: (1) any other clinical disorders besides TNBC were observed; (2) any treatments, such as oral durgs and intravenous injection recevied within 3 months before the present study; (3) and with family history of certain malignancies. Based on the staging criteria established by AJCC, stage I–IV included 19, 20, 18, and 13 cases, respectively. The Ethic Committee of First Affiliated Hospital of Zhengzhou University Hospital and Henan Cancer Hospital approved the present study before the enrollment of patients. All patients signed informed consent.

### Cell lines and transient cell transfections

Hs 578T (Sigma-Aldrich, U.S.A.) and BT-549 (ATCC, U.S.A.) cell lines were used in the present study to perform all the *in vitro* cell experiments. Cells of both cell lines were cultivated with RPMI-1640 Medium (10% FBS). Cell culture conditions were 37°C, 95% air, and 5% CO_2_.

To establish NRON and snaR expression vectors, full length NRON and snaR cDNAs were inserted into pcDNA3.1 vector. The vector construction service was provided by Sangon (Shanghai, China). For transient transfection Hs 578T and BT-549 cells were cultivated overnight to 70–80% confluence. Lipofectamine 2000 reagent (Invitrogen, U.S.A.) was used to perform all cell transfections with 10 nM vector. Two controls, including negative control (NC), which included cells transfected with empty pcDNA3.1 vector, and control (C), which included cell without any transfection but were treated with lipofectamine 2000 reagent, were included in this experiments. Subsequent experiments were carried out at 24 h after transfections.

### RT-qPCR

All total RNA extractions from tissue specimens as well as Hs 578T and BT-549 cells were performed using Ribozol RNA Extraction Reagent (Thomas Scientific). Following reverse transfections performed using Applied Biosystems™ High-Capacity cDNA Reverse Transcription Kit (Applied Biosystems, U.S.A.), all qPCR reaction systems were prepared using qScript One-Step RT-qPCR Kit (Quantabio, U.S.A.) to detect the expression of NRON and snaR with the endogenous control of 18S rRNA. The qPCR reactions were performed in triplicate manner and data were analyzed using the 2^−ΔΔ*C*^_T_ method.

### Cell proliferation assay

Hs 578T and BT-549 cells were harvested at 24 h after transfection. Cells were mixed with RPMI-1640 Medium (10% FBS) to prepare single cell suspensions with a cell density of 4 × 10^4^ cell per ml. A 96-well cell plate was used to culture single cell suspensions (0.1 ml per well). To monitor cell proliferation rates, 10 μl CCK-8 solution (Sigma-Aldrich) was added into each well every 24 h until 96 h. After that, cells were cultured for further 3 h, following by the addition of 10 μl DMSO. Finally, OD values at 450 nM were measured to reflect cell proliferation.

### Statistical analysis

All least three biological replicates (3–6) were included in each experiment to make sure the data were solid. Paired *t* test was used to analyze the differences between TNBC and non-cancer tissues. ANOVA (one-way) and Tukey’s test were used to analyze differences among different clinical stages and cells in different transfection groups. The linear correlation between NRON and snaR expression was analyzed by linear regression. *P*<0.05 indicated a difference with statistical significance.

## Results

### NRON was down-regulated in TNBC and affected by clinical stages

**Figure 1:**
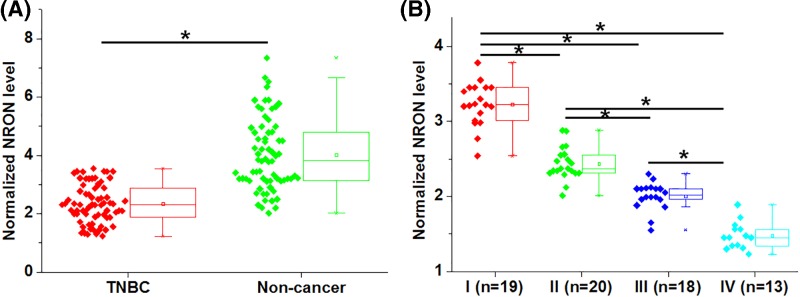
NRON was down-regulated in TNBC and affected by clinical stages Analysis of NRON expression in TNBC tissues and adjacent non-cancer tissues by paired *t* test showed that NRON was significantly down-regulated in TNBC tissues comparing to non-cancer tissues (**A**). Analysis of NRON expression in TNBC tissues among patients with different clinical stages showed that NRON expression levels decreased with the increase of clinical stages (**B**), (*, *P*<0.05).

NRON expression was analyzed by performing RT-qPCR and expression data were analyzed by paired *t* test. The results showed that NRON was significantly down-regulated in TNBC tissues comparing to non-cancer tissues (Figure 1A, *P*<0.05). The expression data of NRON in TNBC tissues were further compared between patients with different clinical stages by ANOVA (one-way) and Tukey’s test. In was observed that NRON expression levels decreased with the increase of clinical stages.

### snaR was up-regulated in TNBC, affected by clinical stages and inversely correlated with NRON

**Figure 2:**
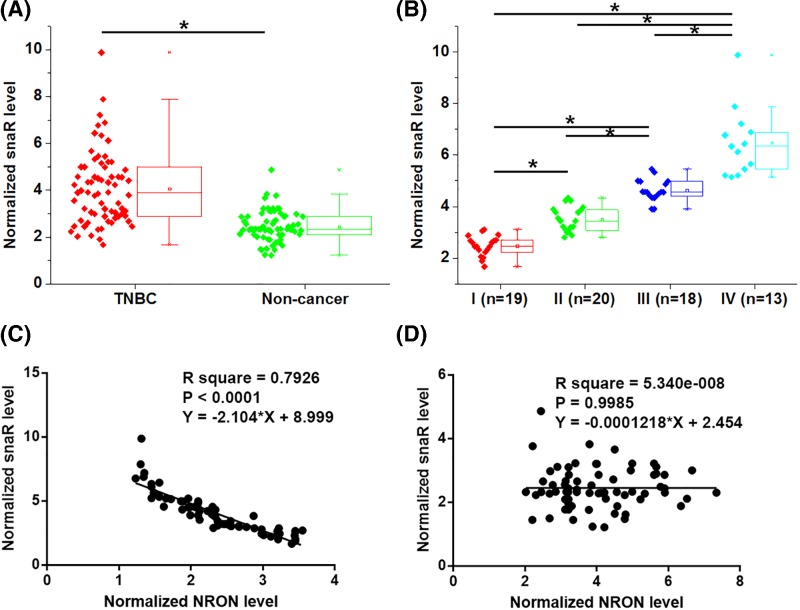
snaR was up-regulated in TNBC, affected by clinical stages and inversely correlated with NRON Analysis of snaR expression in TNBC tissues and adjacent non-cancer tissues by paired *t* test showed that snaR was significantly up-regulated in TNBC tissues compared with non-cancer tissues (**A**). Analysis of snaR expression in TNBC tissues among patients with different clinical stages showed that snaR expression levels increased with the increase of clinical stages (**B**), (*, *P*<0.05). Linear regression analysis showed that snaR was inversely correlated with NRON in TNBC tissues (**C**), but not in non-cancer tissues (**D**).

Similarly, snaR expression was also analyzed by performing RT-qPCR and paired *t* test. Different from NRON, snaR was significantly up-regulated in TNBC tissues compared with non-cancer tissues (Figure 2A, *P*<0.05). ANOVA (one-way) and Tukey’s test analysis showed that snaR expression levels increased with the increase of clinical stages (Figure 2B, *P*<0.05). We therefore performed linear regression to analyze the correlation between these two lncRNAs. Interestingly, a significant correlation between them was found in TNBC cells (Figure 2C, R square >0.65, *P*<0.001), but not in adjacent non-cancer tissues (Figure 2D, R square <0.65, *P*>0.05).

### NRON down-regulated snaR to inhibit TNBC cell proliferation

**Figure 3:**
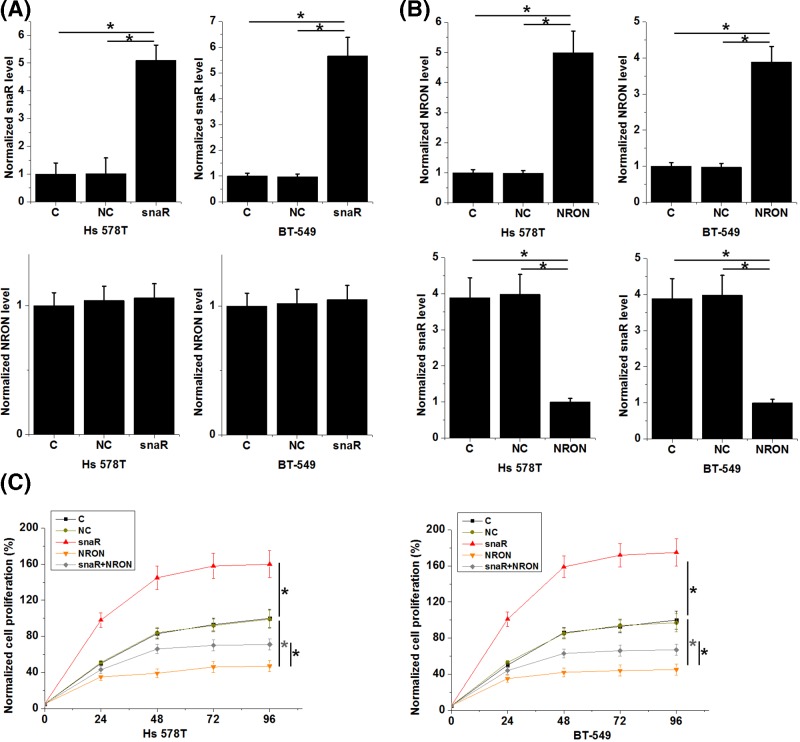
NRON down-regulated snaR to inhibit TNBC cell proliferation Analysis of snaR and NRON expression data showed that, expression levels of snaR (**A**) and NRON (**B**) were significantly up-regulated in Hs 578T and BT-549 cells at 24 h after transfections compared with NC and C. In addition, snaR overexpression promoted cancer cell proliferation (**C**) but did not significantly affect NRON expression (A), while NRON overexpression inhibited cancer cell proliferation (C) and down-regulated snaR (B) (*, *P*<0.05).

The abovementioned data indicated the potential interaction between snaR and NRON. To test our hypothesis, snaR and NRON expression vectors were transfected into Hs 578T and BT-549 cells. Compared with NC and C, expression levels of snaR (Figure 3A) and NRON (Figure 3B) were significantly up-regulated in Hs 578T and BT-549 cells at 24 h after transfections (*P*<0.05). In addition, snaR overexpression promoted cancer cell proliferation (Figure 3C, *P*<0.05) but did not significantly affect NRON expression (Figure 3A), while NRON overexpression inhibited cancer cell proliferation (Figure 3C, *P*<0.05) and down-regulated snaR (Figure 3B, *P*<0.05).

## Discussion

NRON is reported to participate in HIV infection. We investigated the involvement of NRON in TNBC and found that NRON was down-regulated in TNBC and played an tumor suppressive role in TNBC by down-regulating oncogenic lncRNA snaR.

NRON regulate the degradation of tat protein in human cells upon HIV infection [9]. It has been reported that tat interacting proteins play a tumor-suppressive role in different types of cancer [11,12]. Therefore, it is reasonable to hypothesize that NRON may also participate in cancer biology. The present study reported the down-regulation of NRON in TNBC and its inhibitory effects on cancer cell proliferation. Interestingly, our study observed the decreased NRON expression level along with the increase of clinical stages, but NRON overexpression failed to affect cancer cell migration and invasion (only slight inhibitory effect, data not shown). Therefore, NRON may only interact with pathways involved in cancer cell proliferation.

LncRNAs, especially circulating lncRNAs, have been widely used as prognostic and diagnostic biomarkers for cancer due to their altered expression pattern during cancer development [13–15]. However, our study observed a big overlap of NRON expression levels between TNBC and non-cancer tissues. Therefore, NRON may not be a good diagnostic biomarker for TNBC. Another problem for the use of NRON as a TNBC biomarker is that we did not detect NRON in the plasma of most TNBC patients. This is possibly due to its down-regulated expression in this disease.

It has been well established that lncRNAs play their roles by regulating gene expression, such as post-translational regulation, translational regulation, protein degradation, and epigenetic modifications [16–17]. Recent studies also observed that lncRNAs may serve as sponge of miRNAs to inhibit their functions [18,19]. Interestingly, we observed that NRON was likely an upstream snaR in TNBC. However, the mechanism is unknown. We speculated that NRON may indirectly interact with snaR due to the lack of correlation between NRON and snaR in non-cancer tissues. Our preliminary data showed that NRON cannot methylate snaR. NRON may interact with RNA degradation pathways, or certain miRNAs to promote the degradation of snaR. Our future studies will try to elucidate the details of the mechanism.

In conclusion, NRON was down-regulated in TNBC and NRON overexpression may inhibit TNBC cell proliferation by down-regulating snaR.

## Availability of data and materials

The analyzed data sets generated during the study are available from the corresponding author on reasonable request.

## Abbreviations

C: control
NC: negative control
TNBC: triple-negative breast cancer
